# Can psychiatry hinder intersubjectivity? A phenomenological critique of the biomedical conceptualization of anomalous experience

**DOI:** 10.3389/fpsyg.2025.1505028

**Published:** 2025-07-02

**Authors:** Sabina Wantoch

**Affiliations:** Department of Philosophy, The University of Sheffield, Sheffield, United Kingdom

**Keywords:** phenomenology, phenomenological psychopathology, critical phenomenology, epistemic injustice, anomalous experience, intersubjectivity, intentionality, critical medical humanities

## Abstract

This study examines the phenomenon of anomalous experiences. The term ‘anomalous experience’ refers to experiences often described as hallucinations and, more broadly, to the experiential dimensions of what is commonly referred to as psychosis. I present a critical analysis of the dominant clinical conceptualization of anomalous experience, which frames it as a ‘pathology of the mind’, by focusing on how this assumption is experienced intersubjectively. Drawing on Ratcliffe’s (2017) account of how intersubjectivity is implicated in anomalous experience, I argue that the psychiatric conceptualization of such experiences may obstruct intersubjective processes for those who undergo them. I suggest that this pathological marker, through its underlying assumptions and institutional practices associated with it, can give rise to a certain kind of relationality, characterized by an affective tone that excludes individuals from the shared interpersonal dynamics typically structuring experience in relation to a shared reality. Consequently, the psychiatric conceptualization of anomalous experience may play a role in the constitution of experiences of the kind that it seeks to erase. This implicates phenomenological psychopathology to question the starting assumptions that it takes as a given, direct picture of reality. Phenomenological psychopathology often adopts a pathological conceptualization of anomalous experience as its starting point, taking psychiatric concepts as given. I suggest that the discipline consider its own role, phenomenologically, in the multidirectional interactions that take place between anomalous experiences and the ways they are conceptualized and responded to. I propose that beginning with the direct experience itself, rather than its pathological association (and all the affective baggage this entails), would represent a progressive direction for the future of phenomenological psychopathology. This points toward critical phenomenology and critical phenomenological psychopathology.

## 1 Introduction

“Phenomenology offers psychopathology an approach that looks at the lived experience of the patient without presuppositions and preconceptions.”

What if really looking at the lived experience without presuppositions and preconceptions called into question the disorder itself – the starting point? Phenomenological psychopathology traditionally works within the boundaries of disorders. However, phenomenology may also shed light on the limits of such boundaries through nuances relating to the institutional and practical applications of the psychiatric discipline and how this may shape our understanding of the very experiences considered psychopathological. I suggest that the affectivity of psychiatric norms can affect the very structure of the experiences that they aim to capture. This highlights the significance of the social sphere in matters of phenomenology. Here, I refer to how society is organized, through, for example, institutions, bodies of knowledge, and power relations, which may affect how we experience the ‘interpersonal field’ by shaping certain kinds of interpersonal relating. I take our interpersonal relationality to be integral to the structuring of experience. Here, I argue that the social ‘field’ of psychiatry, through psychiatric conceptualizing of anomalous experience, may affect the structuring of experience for those people who are its subjects. Thus, this social ‘field’ is not impartial to the kinds of experiences that it aims to capture and respond to. Rather, it is involved with them at the phenomenological level – the level of the structure of experience. This study presents a mechanism to explicate this suggestion.

This introduces the phenomenological concept of intersubjectivity, which refers to shared experience. This is the basic sense in which we share what we perceive subjectively, with the world being shared with others who also experience it as experiencing subjects. This marks our experience of what we consider to be a spatiotemporal reality as one that others can share. We constantly regulate one another’s perceptual experiences through our interactions and our responses to our shared world. This separates perceptual experience from other modalities of experience that others do not have access to. This brings us to another necessary concept for this study: intentionality. ‘Intentionality’ refers to the defining feature of consciousness, namely that it is directed – we are conscious ‘of’ something. We are aware of x – whether through perceiving, imagining, remembering, or otherwise, ‘x’. These modalities are intentional states, states of directed-ness or about-ness, and represent the relationship between the subject and what is being experienced. I will examine intentionality in terms of its relationship with intersubjectivity–indeed, the ‘role’ that intersubjectivity plays in intentionality–and how this relationship may be constituted in experience considered ‘anomalous’ with respect to its (dominant in the Anglo-West) intersubjective context of psychiatry. I suggest that phenomenological psychopathology could do with being more sensitive to the phenomena at hand, with an awareness of the ways that psychiatric diagnoses and all the institutional, phenomenological, and epistemic ‘baggage’ that they carry for the client may affect this relationship between intersubjectivity and intentionality, thus affecting the very phenomenon of anomalous experience.

This argument follows Ratcliffe’s (2017) conception of anomalous experience, which theorizes the dependence of intentionality on the experience of a shared world–intersubjective experience. There is also a wider phenomenological tradition that, more broadly, holds intersubjectivity as fundamental to intentionality and applies this connection to psychopathology (Fuchs, 2015; de Haan, 2010). There is also literature from the critical phenomenology field whose arguments are motivated by observations of how power moves through the world and how this affects marginalized experience–and, importantly, the structure of experience on the level of intentionality (Guenther, 2013). I am also motivated by similar observations. In addition to utilizing the traditional phenomenological approach of interrogating how it is that I can have experience (what are the necessary conditions for my intentionality), I am also compelled to make this connection through my own experience of how power moves through the world and can affect the *way* that one experiences in general. I am interested in the affectivity of power relations and how they are maintained through institutional norms and practices, as well as how this affectivity can pervade our relationality and, thus, our intentionality if we acknowledge the interdependence of intersubjectivity and intentionality. Once we take this seriously, it compels us to critique the assumptions underlying our phenomenological approach. In the case of phenomenological psychopathology, this means critiquing pathological labeling itself.

This study asks and proposes a response to the question: What can phenomenology critically offer the study of psychopathology in regard to anomalous experiences? This concerns the phenomenological affectivity of the dominant psychopathological conception of anomalous experience as symptoms of disorder. I am interested in the affect that this has regarding the intersubjective field of the person having such experiences and how this may interfere with the experiences that phenomenological psychopathology aims to examine and treat. Therefore, in answering this question, I propose a mechanism that suggests there may be a feedback loop between the psychiatric framing of anomalous experiences and those experiences themselves. This suggests that, rather than being an impartial observer, the psychiatric framing of anomalous experiences is phenomenologically involved with these experiences at their constitutive level.

Phenomenology’s approach to examining experience in the most direct way possible, without presuppositions and preconceptions, questions the psychiatric framings of psychopathology, which is indeed what this study aims to interrogate in relation to anomalous experience. For this reason, the study focuses on a type of experience rather than a disorder. I focus on anomalous experiences because they are conceptualized as a symptom of disorder, and I am interrogating that assumption. Thus, I use the term ‘anomalous experience’ to refer to experiences that are often called ‘hallucinations’ and often interpreted as positive symptoms of schizophrenia or psychosis. My starting point, however, is not this interpretation but a ‘type’ of experience – a vague and heterogenous type but roughly characterized as experiences whose content is felt viscerally, with some qualities of perception, but which do not correspond to a perceptual object shared by others. It is these experiences that I refer to as the phenomena at hand whilst acknowledging that this is a vague category. For instance, can anything ‘strange’ and not otherwise explained count as ‘anomalous’? It is these periphery cases, as well as more extreme cases, that I am interested in, particularly in regard to how they get responded to and conceptualized. Since my focus is on their dominant framing through psychiatry and its affect on the experiences, it is important to note that even less disruptive anomalous experiences are commonly associated with the presence of pathology or the risk of it. Thus, I am interested in any experience which may get ‘caught’ in the slippery extension of ‘anomalous experience’.

## 2 Phenomenological conception of anomalous experience

Ratcliffe (2017) has an in-depth phenomenological account of anomalous experience, which I will draw on. In addition to being an extensive and nuanced account, it draws on a phenomenological tradition that links the fundamental nature of experience with the experience of the interpersonal world by taking intersubjectivity as fundamental to subjectivity. Since I am interested in the interactions between the interpersonal world in regard to anomalous experience (how it is conceptualized as pathological) and anomalous experience itself, a phenomenological account that takes seriously the role of the interpersonal world on the structure of subjective experience appeals in its potential to provide a nuanced picture of these interactions. I am also drawn to Ratcliffe’s account because it is a rich account that seeks to characterize how these experiences feel, as opposed to the traditional conception of hallucination as simply ‘perceptual experiences in the absence of environmental stimuli’. Ratcliffe draws on Husserl (as well as other classical phenomenologists) and extensively develops phenomenological work in the field, which is a compelling place to start in analyzing phenomenology’s contribution to psychopathology regarding anomalous experiences, as well as how it may be applied in terms of the involvement of the interpersonal world.

### 2.1 The structure of intentionality is dependent on the interpersonal

Ratcliffe’s work on anomalous experience highlights a relationship between anomalous experiences and intersubjectivity by constructing a picture of the structure of intentionality, which frames the intersubjective nature of experience. Here, the ‘structure of intentionality’ is the structure that is at the core of self-consciousness, the structure that makes it possible that we can be in an ‘intentional state’. ‘Minimal self’ also broadly refers to the same ‘thing’ here, the ‘thing’ that makes it possible for us to have intentional awareness. Ratcliffe’s account of this is a rich one that includes how we recognize the kind of intentional state we are in, as well as how it is that there are certain types of determinable intentional states with distinct natures. ‘Minimal selfhood’ is the pre-reflective self-awareness of experience – that you are experiencing *something*–and Ratcliffe is committed to the view that this must be comprised of an awareness of not just being in an intentional state (the awareness that you are experiencing something) but also of an awareness of *the type* of intentional state that one is in (Ratcliffe, 2017, p. 18). Here, we are referring to intentional state types, such as imagining, remembering, and perceiving. The structure of intentionality is the hypothesized ‘structure’ that is responsible for creating these determinable types of experience and the self-conscious awareness of being in them. Ratcliffe argues that such a rich account is implied by our very pre-reflective experience. I will briefly summarize it below.

Starting with our pre-reflective awareness, even at such a minimal level, our awareness is of something; pre-reflective awareness involves being aware of ‘p’. Being aware of p is being aware of its spatiotemporal location; all our experiences are through our locus of space and time, as we are spatiotemporally located in the world.^1^ Being aware of p’s spatiotemporal location involves being aware of p in a particular way. This is to be aware of what kind of intentional state we are in: are we perceiving, remembering, or imagining p? The idea is that we know how we are aware of p in terms of whether we are aware of it now, in front of us in the same space, if we are aware of where it once was (remembering), or if we are aware of p in a way that is less connected to any spatiotemporal location (imagining). While the distinctions between types of intentional states are not clear-cut, he claims there is a certain ‘sense’ that comes with being in a specific intentional state that contributes to the experience of that intentional state (Ratcliffe, 2017, p. 21). Ratcliffe also differentiates between the actual intentional state type that one is in and, on the other hand, an experience that is characteristic of an intentional state type, as well as having the ‘sense’ of an intentional state type. This is because it is possible to be mistaken about the kind of intentional state that one is in, e.g., a false memory that is an imagining. This preserves a ‘connection to reality’–that we can be wrong about our experiences and that we can be wrong about their modality as well.

Ratcliffe presents our awareness of perception, that we are perceiving rather than imagining p, as constituted by an awareness of what is ‘here and now’ as ‘real’ for everyone to see. The sense of something being here and now, in front of me, comes with a sense that it is also available to others. What is here and now is what can be corroborated by other beings perceiving the world as well. I know I am perceiving p because I have a sense that if someone else were to be here, now, with me, sharing my spatiotemporal location, they would also perceive p. In this way, other people implicitly validate what we perceive. This occurs in a constant, implicit, regulatory manner, where we anticipate our own and others’ perceptual experiences, and this feedback influences our own anticipatory perceptual experiences, shaping our experience of the world around us. For example, when I walk onto a bus, I anticipate that there will be a driver waiting for my money and seats laid out in a certain way, as well as stairs. This anticipation is interpersonally regulated; it is based on others’ reactions and validations, as well as what we learn as we navigate a social world. I know the seats will all be there–as opposed to in my head–because people will be sitting on them, and I have learned it will be this way through previous experience or social conventions, and other people will be acting as if they are there, responding to them. And the driver needs to take my money. If the bus is empty, I am aware that other people could get on and sit in the seats; they are things that exist in a shared way–experienceable to others in time and space, just as they are to me.

Indeed, it is ‘shared projects with other people’ (Ratcliffe, 2017, p. 147) that structure perceptual experience in that they dictate what we focus our attention on. This includes things like plans made with others, such as meeting up with a friend, having a conversation with a teacher, or eating dinner with the family. We do not usually question the sense of realness of the social world (the true existence of other people) like we might question something we see or remember; rather, we look to others for corroboration and validation. This implicit and habitual looking to others for corroboration of perceptual experience presupposes a basic, deep-rooted trust in their existence as ‘real’. Although we may question people’s intentions or interpretations of social dynamics, we rarely seriously question the basic sense that the interpersonal is our reality: that I need to get on the bus and interact with the driver to get to work, for example.

This is what Ratcliffe calls ‘habitual trust’: the basic trust that the world is real and that others also share in this realness. This gives perceptual experience a sense of realness and references a corroborable shared world with others when we reference reality. In this way, habitual trust underscores our awareness of other ways of experiencing in terms of how they feel in relation to the feeling of realness and sharedness. This trust in reality as shared, gives perception its characteristic feel as perception, and thus other intentional state types their characteristic feelings in terms of how they are felt against a general sense of realness associated with spatiotemporal location and the affective anticipation of others.

Habitual Trust is affective, an attitude that is naturally ingrained in our lives. Because it is bound up with ‘affective anticipation’ that coheres and structures the world around us, reliant upon other people corroborating those anticipations, this trust is reliant on other people, both developmentally (as we first learn about the world through other people’s experience of it) and constitutively–as we need others to provide this affective regulatory relatedness. Other people have a ‘stake’ in our reality; they corroborate or correct it in a minimal way all the time. This relatedness is an integral part of how we experience anything, as it shapes a sense of intersubjective realness. This means that, crucially, our awareness of which type of intentional state we are in is reliant (both developmentally and constitutively) on other people through this intersubjective trust in reality: a “habitual confident immersion in the world” (Ratcliffe, 2017, p. 156).

Other people play such a constitutive role in our structuring of experience because while the world speaks to us in very real, sensory ways, it is other people who corroborate and shape those sensory ways to be what they are, with all their significance for us in living in a world with others. When I perceive that my bike is outside the window, I perceive it as part of the here-and-now world, and I perceive it as something perceivable to other people. Other people are not like my bike; they can perceive my bike as I can, and the thrust is that perceiving my bike would be, look, and feel very different if it were not implicitly tied to a pre-reflective awareness that it is perceivable to others. Availability to others is a characterizing factor of how perception feels: it is tied into its anticipatory structure, and the anticipation of what comes next is bound up with the presence or potential presence of other perceiving subjects. Ratcliffe (2017, p. 123) references the work of Husserl to explain: “the phenomenological difference between encountering something as really there, independent of my own perspective on it, and experiencing it as self-generated is constituted by a sense of whether or not it is actually or potentially accessible to others.” The idea that our sense of reality is upheld by ongoing shared processes with others and the world is also reflected in work in the enactivism tradition, applied to experiences considered psychopathological (Fuchs, 2015; Fuchs, 2020).

### 2.2 Anomalous experiences

Habitual trust is “inextricable from a way of being immersed in the interpersonal world and consequently vulnerable to certain kinds of disruption” (Ratcliffe, 2017, p. 36). Ratcliffe conceptualizes experiences characterized as ‘hallucination’ (what I refer to as anomalous experiences) as experiences that are constituted by disruptions to the structure of intentionality – when the characteristic affective profiles of intentional states get blurred or lose their characteristic form, giving way to experiences of a form somewhere in between the usual intentional state types. Anomalous experiences, on this account, are constituted as changes to the structure of intentionality, where the sense of reality is felt in an unattached way to other people and the social world. This could be general, or it could be more specific and temporary, concerning localized experiences. Anomalous experiences are characterized as experiences that are ontologically likely to be imaginings, though felt with the sense of perception, and phenomenologically much more complex, as these kinds of changes constitute all sorts of phenomenological nuances – whether locally concerning specific experiences or globally concerning one’s general experience. The consensus world may occupy a different function in relation to how the person structures their experience; ‘the flagship of reality’ is placed elsewhere, unattached to patterns of anticipation and corroboration with other people.

I avoid conceptualizing anomalous experiences as necessarily ‘mistaken imaginings,’ and I do not take this characterization to be exhaustive since I want to remain open to all sorts of constitutions and ontological statuses of such experiences. I consider anomalous experiences to be different types of experiences, marking a distinct kind of connection to the consensus world and intersubjective reality, but without necessarily being defined as defects in an ideal structure of intentionality.^2^

The important thing here is that habitual trust connects us to a shared reality with others, and this structure influences the way we experience the world. The detachment or breakdown of habitual trust means that ‘reality’ will feel different, and what is perceived or seems like perception will also feel different. Because this relationship is constitutive, shifts to the structure of intentionality may be caused by interpersonal events. Traumatic experiences may subvert the sense of reality, making it misaligned with that of others, such that they are not sources of corroboration but rather of threat. If not in such a global way, this might happen locally and episodically, concerning how the traumatic experience in question is integrated into a person’s structure of intentionality through remembering it: it may not be remembered like any other perceptual experience since there was something about it that subverted the characteristic sense of what perception is. An interpersonal event that radically subverts one’s general attitude toward others is an experience that contains perceptual contents that may have uprooted the person’s very sense of what perception is since one’s general attitude toward others constitutes part of this sense. This explains how certain interpersonal experiences (for example, abuse, threat, and trauma) can have an effect that transcends content: they can alter the very form in which the experience is remembered or how one perceives it more generally (Ratcliffe, 2017, p. 132).

Therefore, intentionality is constitutively (and developmentally) dependent on intersubjectivity. Shifts or ruptures in one’s ability to feel intersubjective trust will constitute shifts or ‘ruptures’ in the way they structure their experience and feel the kinds of intentional states they are in. We might refer to this as a form of ‘transcendental intersubjectivity’. Guenther (2013) study is relevant in this context, as she charts how the experience of solitary confinement of prisoners constitutes an unraveling of intentionality–of the transcendental structures of consciousness–through depriving a person of relationality. She argues for the transcendental nature of intersubjectivity from this perspective.

Even if we do not take Ratcliffe’s (2017) rich account of the minimal self and its dependence on others, it is clear that having a sense of intersubjective trust is still necessary for shared experience. Therefore, experiences that threaten such trust will have a significant impact on one’s ability to coexist in a shared world with others. In regard to anomalous experiences and phenomenological psychopathology, this calls into question the interpersonal social contexts through which we navigate, relate to and respond to anomalous experiences. I will now turn to look at psychiatry and its wider reverberations in society through its institutional practices and assumptions related to people with anomalous experiences. Here, I will specifically focus on its conceptualization of anomalous experience at the root of these practices and assumptions, which is relevant to phenomenological psychopathology. I will suggest ways in which this may shift or rupture a person’s relationality and habitual trust to call into question phenomenological psychopathology’s reliance on psychiatric diagnosis as its starting point. This is particularly relevant to anomalous experiences and their diagnostic markers, but the suggestion may also be applicable to other disorders. The suggestion is that pathological assumptions about anomalous experiences may lead to different kinds of relationality for individuals experiencing these phenomena in terms of how they interact with clinicians as well as the broader world due to the ‘baggage’ of such assumptions, which I will explain below. I aim to make suggestions and propose a mechanism for how this may happen, prompting critique of the common approach in the phenomenological psychopathology tradition of starting with diagnostic labels as a direct representation of objective reality.

## 3 Psychopathological conception of anomalous experience

Here, we are interested in what the psychiatric conceptualization and response are like at the level of intersubjectivity. What is its affectivity as an intersubjective field? What kinds of relationality does it instigate and uphold through its processes of diagnosing, conceptualizing and intervening with anomalous experiences? I am not necessarily focused on individual relationships with psychiatrists, as these are extensively varied, but on the wider aspects of this dominant (and legally enforced) way of conceptualizing and responding to anomalous experience as pathology: how it shapes the world and shared processes in relation to people having these experiences. I focus on three aspects of this: implied diminished epistemic agency, the phenomenon of being ‘marked’, and alienation from intersubjective processes. I will briefly outline these to propose that psychiatry itself may play a role in disrupting habitual trust.

Anomalous experiences are generally interpreted as symptoms of an essential condition, such as schizophrenia, type I or II bipolar and various other psychotic disorders. By ‘essential condition’, I am referring to the way in which these disorders are framed as long-term or chronic conditions that stay with a person and that they will consistently have to manage; they are framed as something relating to that person’s fundamental existence in the world. The presence of hallucinations and delusions (occurring persistently and with ‘reduced functioning’) is enough to be DSM-diagnosed with schizophrenia (Maiese and Hanna, 2020). Even outside of formal medical processes, common attitudes associate such experiences with a loss of reality due to pathology or the start of this process. Anomalous experiences are framed within an affect of threat and urgency: they must be responded to quickly, as they are signs of the mind’s pathology and carry great risk. What I am drawing attention to here is the essentializing aspect of pathological diagnostic markers; they concern a person’s whole existence and their ability to know reality, to partake in shared reality as an epistemic agent. Their sense of reality is assumed to be compromised, considered to be outside the realms of epistemic capability (or their epistemic capability is at least diminished) – even, and especially, in regard to their knowledge about their own experience. John Hood, a person diagnosed with schizophrenia, sums up the experience of living with this assumption: “When it comes down to it, there’s no greater stigma than the client thinking that his mind is diseased” (Luhrmann, 2016, p. 34).

Epistemic harms in psychiatry have been well documented by Crichton et al., 2017, as well as Kidd et al. (2022). Crichton et al. (2017) show that psychiatric patient encounters with clinicians, as well as encounters between perceived psychiatric patients (or people perceived to be in need of psychiatric intervention) with members of the public, persistently involve assumptions that lead to their testimonies not being believed, leading to cases where the patient’s/ perceived patient’s autonomy is restricted due to untrue assumptions being applied by the clinician. They also argue that in the case of schizophrenia, there are specific assumptions about the disorder involving the patient’s epistemic agency that lead to increased testimonial injustice; “the stereotype of ‘split personality’ is, of course, a perfect example of a fragmented epistemic self with whom one cannot effectively engage either socially or epistemically” (Crichton et al., 2017). My argument is that assumptions of diminished epistemic agency are associated with having anomalous experiences through their association with disorders such as schizophrenia or psychotic disorders and that these assumptions are institutionally entrenched. They lead to – not just these people’s testimonies being denied in everyday encounters – but a wide-ranging denial of their contributions to shared knowledge creation, including their contributions to knowledge about their experiences. Importantly, this assumption is applied across the board in the presence of anomalous experience rather than on a case-by-case basis.

Psychiatric diagnoses and the ways that a person is related to them, in accordance with such stigma, affect one’s epistemological standing against others. Here, I suggest that this has a phenomenological significance. Regarding anomalous experiences and the assumption of diminished epistemic agency that accompanies them, I suggest that this may challenge a person’s ability to participate in the shared processes of corroborating each other’s perceptions, which are necessary for sustaining habitual trust in the shared world. This is because there is an existential component to intersubjectivity, also referred to as the ‘i-thou’ level of interpersonal intersubjectivity.^3^ It marks that the inherent notion of habitual trust outlined in pre-reflective intersubjectivity is an awareness of others as subjects. Their subjective awareness of reality, which is intertwined with our own through shared processes of affective anticipation, requires us to relate to them implicitly as epistemic agents able to share in these processes together. If a person is consistently associated with someone who lacks epistemic agency, then this may pervade their relationality in general and their access to intersubjective processes necessary for developing habitual trust. I will further develop this below.

By acknowledging the affective component of shared knowledge-making practices and norms that epistemic injustice literature interrogates, we see how intersubjective processes are influenced by power dynamics and marginalization because this is how the social world is structured. Here, I am referencing how we implicitly relate to one another as subjects of a shared world, able to corroborate or challenge our perceptions as a requirement of shared intersubjective practices. If a person is habitually related in a way that undermines this requirement, it may affect their relationality in general, thereby affecting the level of their structure of intentionality. ^4^ I suggest below that persistent threats and alienation regarding others may constitute processes that unravel habitual trust and change how a sense of realness is felt.

Legghio (2013) argues that while it is often claimed that psychiatry silences its patients, this analysis does not go far enough because these people are not only silenced but are also made hyper-visible in being targets for coercive responses. Rather than being silenced or not noticed, being denied as knowers means that their way of being and experiencing and knowing the world is actively denied as illegitimate, which can have dramatic consequences:

“Dismissed as incompetent, the psychiatrized person cannot get their knowledge, the content of their experiences, or their ways of knowing recognized and heard as legitimate. Alternative experiences of reality-defined as “psychosis” or “hallucinations” - become the rationale for the denial of their legitimacy as a knower. Rendered incompetent persons are disqualified as legitimate knowers and lose their epistemic agency, specifically losing their ability to speak on their own behalf and to be heard on their own terms and in their own styles.” (Legghio, 2013, p. 126).

Samra (2023) argued that the disorientation that occurs in a person’s sense of self following consistent doubt about their perceptions of reality can be categorized as the harm that occurs in both the ontological domain (regarding their representations of reality) and the epistemic domain. This demonstrates the ‘weight’ that I am referring to: that such asymmetry between the person and others, amongst a backdrop of real or threatened institutional coercion, may constitute ontological shifts in one’s sense of reality.

What I am drawing attention to is the extrapolation that takes place, from a kind of experience to an assumption of denied epistemic agency. Whilst there are cases in which it is appropriate to exercise caution regarding the contributions to shared knowledge-making of a person with anomalous experiences, where we may question their interpretation of an experience, this should be judged on a case-by-case basis. I am interested in how this practice applies in a generalized way through the association of such experiences with pathological assumptions. Though there will be many cases where someone’s credibility as a knower is compromised as a result of their mental health, it is not the case that the assumption of distrust should be extrapolated from the mere presence of anomalous experience. The point here is that the pathological framing of anomalous experiences generally assumes a denial of these individuals’ epistemic ability, which may amount to a different kind of relationality with the world if they are consistently excluded from shared affective processes necessary for habitual trust and thus cohering a sense of reality with others. It is this across-the-board extrapolation that may leave people vulnerable to being excluded from such processes, as they are related to as those who cannot access this sharedness. The phenomenon of ‘clinical insight’ demonstrates the fixed degree to which this extrapolation takes place. Having ‘clinical insight’ means a patient agrees with the pathological framework regarding their own experience. It is considered insight into their condition–knowledge–but when a patient appeals to any other alternative framework to understand their own experience, this is considered incorrect and is taken to be evidence that a person is ‘more ill’ – thus subjugating them to the pathological framework. If a person has a different interpretation of their experiences, that does not see them as symptoms of a disorder, then this is taken to be a sign of lacking in clinical insight, which is seen as a further symptom of illness (Roe et al., 2008, p. 2). Alternative frameworks are ejected and read as symptoms of being more ill; possible readings of meaning or value into the anomalous experience are denied as delusional. I state this to highlight the phenomenological weight of such an assumption and how it sets up an asymmetrical relationship between the person having the experiences and the intersubjective community of people who relate to and respond to them, where they are assumed to be unable to access shared reality.

Ahmed’s (2006, 2007) account of disorientation in relation to living in a racialized world is applicable here in terms of the phenomenon of ‘marking’. It denotes how race ‘marks’ one out, which leads to all sorts of othering treatment of the person by others, that constitutes a wide-spread affect of disorientation – of ‘losing one’s way’. Ahmed describes disorientation as an experience of being ‘out of sync’ with the space in which they try to move through because of the way that such marking ‘stops them’. For example, she talks about this in relation to having a Muslim name and how this ‘marks’ her in certain ways–as a “could be terrorist”–by institutions and authorities in ways that people without this marking are not. This demonstrates the affectivity of a person being marked out in a generalized way that is ‘unlimited’. This label follows them around and holds a certain meaning for institutions and other individuals, as people generally follow institutional practices, biases, and assumptions. This experience of moving through the world in such a way is described by Fanon (1986, p. 83) as being “surrounded by an atmosphere of certain uncertainty.” This atmosphere applies across the board, outside of acutely threatening spaces, due to the generalized affect of threat that being marked in such a way constitutes:

“Can I use this toilet? Why did that police car slow down as it drove by? Why are the diners at the next table staring at me? Why is this security guard following me as I shop? For both Fanon and Ahmed, no space is entirely free from the threat of being stopped. As Ahmed emphasizes, the threatening character of these spaces means that “[t]hose who get stopped are moved in a different way” as they find their way through the world (Ahmed, 2006, p. 162); they are never allowed to fully extend and take shape within everyday contexts of betweenness.” (Krueger, 2021, p. 26).

Ahmed’s experience of having a Muslim name may function similarly to the experience of having a diagnostic label of schizophrenia, for example, in how it is institutionally attached to a person, legitimizing the possibility of coercive responses and the likelihood of their behavior being interpreted in such a way, and shifts their relationship to those around them through how they are seen, habitually.^5^ The person is made visible in a way that they cannot control or contribute to, as their actions may be interpreted according to a risk framework that applies due to the diagnostic marker. These practices are conducted based on criteria such as harm to self or others, indicating that their justification involves more than just broad principles and are considered on an individual, case-by-case basis. However, my point here is to show that, generally, they are carried out according to a framework that pathologizes these experiences on a continuum that could lead to coercive intervention.^6^ The threat of being responded to in ways that severely constrain one’s physical freedom is present just through this marking and its essentialized framework. In addition to a denial of epistemic agency, there are institutional and relational threats to a person that are implications of this assumption. Therefore, the shared world may become a place of ‘certain uncertainty’, threatening yet inaccessible and distant, which only perpetuates the threat in a ‘floating’ manner. This may amount to an ongoing unraveling of habitual trust as a person’s relationality (including their connection to intersubjective reality) shifts shape, and other kinds of experiences may hold more viscerality, comfort, or scaffold a sense of kinship.^7^ This suggests that psychiatry, through pathological markers and their affectivity, may play an ontological role in sustaining or developing anomalous experiences over time through shifts in relational processes that begin with diagnostic labels or even the threat of them.

This affectivity is more obvious at the level of incarceration, forced medication, restraint and solitary confinement that occur as psychiatric responses since these are clearly traumatic experiences that may subvert one’s sense of relationality in general – especially considering that such events may be a person’s only contact with other people since they are incarcerated or even solitary confined.^8^ Here, and in relation to phenomenological psychopathology, I propose a mechanism by which diagnosis may also disrupt habitual trust. This is achieved through the ‘phenomenological weight’ outlined above, which marks a person against shared knowledge-making procedures, the shared world in general, as well as in relation to wider stigma and the possibility, and thus the threat, of incarceration.

I will now turn to phenomenological psychopathology to question its practice of starting with diagnostic markers as a direct picture of reality that is observed phenomenologically. The above argument calls for consideration of the role of diagnostic markers, the affective weight they carry for the person to whom they are applied, in the phenomenon itself: anomalous experience. I suggest that there are ways to analyze the experiences phenomenologically without holding the assumption that they are symptoms of a disorder or, regarding phenomenological psychopathology, symptoms of defects in the structures of experience.

## 4 Implications for phenomenological psychopathology

Phenomenological psychopathology has a tendency to begin with diagnostic labels and interpret the experiences associated with these phenomena as the nature of such conditions. It often interprets the conditions as disruptions or changes to the structure of experience – to ‘normal’ intentionality. If intentionality is dependent on intersubjectivity, then these changes in intentionality will be vulnerable to certain kinds of interpersonal experiences and relationships that may themselves subvert the processes of shared anticipation and corroboration that comprise our shared world-making, thereby structuring our experience of the world. This calls for a critical examination of how we relate to such experiences and how psychiatric narratives and practices may play a constitutive role in shaping the experiences we are examining through its existence as an intersubjective community that exerts power in the world. This calls us to examine the intersubjective dimensions of our starting assumptions. We should strive to engage in phenomenology and apply it to the distressing or unusual experiences that interest us in a dynamic and responsive manner (or, at the very least, one that is aware of such connections and interrelatedness). By looking closer at the experiences in their most direct form and at itself as an ‘intersubjective field’, phenomenological psychopathology could develop reflexivity and criticality of its own role in the intersubjective dimensions of the experiences it is examining, including and related to its starting assumptions, which are often bound up with the psychiatric marking described. Assuming the experiences to be symptoms of essentialized pathologies and defective structures of consciousness may inadvertently take us away from their phenomenological richness whilst also upholding assumptions that have pervasive affectivity for the people to whom they apply–even if we are aiming to reduce such stigma. I have argued that this affectivity may play a constitutive role in the presence of anomalous experience by marginalizing the experiencers from shared intersubjective processes necessary for intersubjective trust.

This may raise questions about the entire project of Phenomenological Psychopathology itself. Is it reliant on these structures of consciousness as fixed essential structures through which it defines anomalies as disordered, pathological versions of these structures gone ‘awry’? Or can it make space for other versions of these structures? Is it prepared to acknowledge the implications of just how entangled we are, such that social contexts, marginalization and power dynamics can reach down into the way we structure our experience, playing ontological roles in the kinds of experiences that challenge such essential structures? This is a call for critical phenomenology and the possibility of a critical phenomenological psychopathology. Indeed, this may lie outside of the boundaries of phenomenological psychopathology’s commitments.

Central to this conjecturing is the question of whether pathology is central to phenomenological psychopathology. Is it possible to see the structure of consciousness not as an essentialized structure but as fluid and flexible, of which different versions are possible? Similar to the aims of the neurodiversity movement, for example, could phenomenological psychopathology work with different kinds of structures of intentionality rather than aim to ‘fix’ them? If Phenomenological Psychopathology is committed to an essentialized structure of consciousness against which anomalous experiences are defective, then it runs the risk of conceptualizing anomalous experiences as inherently other and outside of shared intersubjective processes. Rashed (2015) argued that phenomenological psychopathology’s focus on radical empathy, which assumes a fundamental otherness of the schizophrenic, presumptively leaves them out of a fair chance of being understood and recognized through shared processes and thus takes us further away from the experience at hand. Similarly, Morgan (2022) has explicated the commitments of Jaspers, regarded as the founder of phenomenological psychopathology, in Jaspers’ conceptualizing of schizophrenia as essentially un-understandable and, therefore, in need of explanation.

“At a fundamental level, Jaspers is arguing that we cannot empathize with the person who is mad. Not only can we not imaginatively transpose ourselves into their life history to grasp the connections from one event to another, but there is also a fundamental breakdown in the immediate empathic grasp of them as expressive beings when we meet them face to face” (Morgan, 2022, p. 22).

This highlights the absolutism inherent in the pathological conceptualization. Whilst such a strong position is not necessarily shared across general psychiatry explicitly, this sheds light on possible assumptions within the phenomenological psychopathology discipline and its framings of anomalous experience. This study argues that the essentializing pathological assumption – its phenomenological weight, rather than some essential failure of the anomalous experiencer – may be interfering with intersubjective processes. In making this argument, I have presented a way of arguing phenomenologically about anomalous experiences while not holding such experiences to be essentially pathological. I have argued that the sense of reality in such experience may be more aligned with forms of experience that are unhinged from mainstream intersubjectivity, but I am not committed to holding that this itself is the sign of a disordered mind or a disordered structure of intentionality. I believe that the possibility of a person being able to trust in intersubjectivity and thus find some sense of access to the shared world lies in our being open to this possibility at a fundamental level. This requires us not to define such experiences from the outset as outside the realms of sharedness or unknowingly push them to the margins through the phenomenological weight of pathological assumptions.

## 5 Conclusion

In addition to gaining insight into experiences categorized as psychopathological, phenomenology can also question the ways that psychiatry’s biomedical model is used to conceptualize psychopathology by drawing attention to the wider feedback loops that may exist between this conceptualization and the experiences themselves. I examine this in relation to anomalous experience, applying Ratcliffe’s (2017) model of anomalous experience, which theorizes the dependence of intentionality and minimal self-experience on intersubjectivity. This interdependence has also been theorized by others across the field and is increasingly used in phenomenological psychopathology. This study aims to suggest ways that, by taking seriously the pervasiveness of intersubjectivity in shaping our experiences, the intersubjective life of those having anomalous experiences may play an ontological role in the development of their anomalous experiences. The intersubjective life of such people is bound up with the psychiatric system and its norms; their experiences are conceptualized as pathological symptoms of a disorder of the mind’s ability to know reality and are responded to as such. I have argued that this assumption and its impact on the world can constitute a different kind of relationality, where these people are excluded from the shared processes that constitute habitual trust, which structures their experience in reference to a shared reality with others. This may play a constitutive role in the phenomenon of anomalous experience. I conclude that phenomenological psychopathology could critically reevaluate its own starting assumptions and remain closer to the experiences at hand as they emerge in the world for people, which challenges the discipline’s reliance on pathological framings of such experiences. These experiences are often entwined in complex and nuanced relationships and contexts that a person exists in, in the world with others, both developmentally and constitutively. Phenomenological psychopathology should consider itself as one such intersubjective context and interrogate the phenomenological weight that its assumptions carry.

## Footnotes

^1^Arguably there are exceptions to this during experiences such as meditation, but here we are concerned with casual experience and prereflective awareness of it.

^2^See Wantoch (2022, pp. 41–60) for an in-depth look at this other framing. The focus is on how we can integrate awareness of anomalous experiences as different types of experience, into the consensus world and our relating with others, through interpersonal spaces that are open to these kinds of experience.

^3^Rodemeyer (2020, p. 234) has argued that there are several levels of the constitution of experience in Husserl’s phenomenology.

^4^Guenther (2017) considers this connection in her analysis of how the phenomenological tradition can influence the literature of epistemic injustice through illustrating how the core of our being is in relation to others; that we make meaning of the world intersubjectively, through an awareness of others as subjects like ourselves. Thus, if others are denying us as knowers, as consciousnesses able to make meaning from the world, then this has ontological and existential ramifications. The latter involves how it has ramifications in regard to something core to our beings as subjectivities. Kidd et al. (2022) also conjecture on combining phenomenological psychopathology and epistemic injustice.

^5^See Luhrmann (2016) for an analysis of John Hood’s experience of moving through the world with the label of schizophrenia: “When I talk to people, I have to say, ‘I am a person with schizophrenia,’ and I do not like that,” and cites “the idea that you have a diseased brain that destroys you” as the greatest stigma of all. Another person with the diagnosis states that being told she is schizophrenic “means that they are not schizophrenic,” highlighting this existential mark of otherness (Luhrmann, 2016, pp. 34–35).

^6^Indeed, when we acknowledge the breadth and depth of epistemic harms related to having a mental disorder, we are open to seeing all the ways that interactions with others may be skewed such that a person ends up being coercively treated in a way that is unfair and uncalled for.

^7^For example, entities that one relates to. I am not making a judgment about these experiences necessarily being ‘bad’ but that psychiatric processes may have played a role here.

^8^I have argued in depth for the affective experience of psychiatrization as one that can disrupt habitual trust and therefore play a role in the sustaining or development of anomalous experience (Wantoch, 2022).

## Author’s note

For the purpose of open access, the author has applied a Creative Commons Attribution (CC BY) licence to any Author Accepted Manuscript version arising.
